# Effects of the COVID-19 Pandemic on the Number of New Dementia Diagnoses and the Quality of Dementia Diagnostics and Treatment

**DOI:** 10.14283/jpad.2024.150

**Published:** 2024-08-10

**Authors:** M. T. Hoang, P. G. Jurado, T. Abzhandadze, S. Mostafaei, M. Mo, M. Åkerman, K. Vestling, C. Chen, H. Xu, M. Eriksdotter, Sara Garcia-Ptacek

**Affiliations:** 1 Division of Clinical Geriatrics, Department of Neurobiology, Care Sciences and Society, Karolinska Institutet, Blickagången 16, 14183 Stockholm, Sweden; 2 Department of Medical Epidemiology and Biostatistics, Karolinska Institutet, Stockholm, Sweden; 3 Department of Clinical Neuroscience, Institute of Neuroscience and Physiology, University of Gothenburg, The Sahlgrenska Academy, Gothenburg, Sweden; 4 Department of Occupational Therapy and Physiotherapy, Sahlgrenska University Hospital, Gothenburg, Sweden; 5 Theme Inflammation and Aging, Karolinska University Hospital, Stockholm, Sweden

**Keywords:** SARS-CoV-2, dementia, drugs, medications, quality of care

## Abstract

**Background:**

Care trajectories were disrupted during the COVID-19 pandemic. However, how COVID-19 influenced the number of new dementia diagnoses, and the quality of dementia work-ups, and treatment is understudied.

**Objective:**

To investigate the change in new dementia registrations, diagnostics, and treatment in the pre-, COVID-19 and post-COVID-19 pandemic periods.

**Design:**

A nationwide cohort study.

**Setting:**

This population-based study used data from the Swedish Registry for Cognitive/Dementia disorders - SveDem, and other nationwide registries in Sweden.

**Participants:**

Persons with dementia diagnosed between 2019 and 2021 were divided into three groups based on the date of diagnosis

the pre-COVID-19 period (01 January 2019 – 29 February 2020), the COVID-19 period (01 March 2020 – 31 December 2020), and the post-COVID-19 period (01 January 2021 – 31 August 2021).

**Measurements:**

Outcomes included dementia diagnostics and treatments.

**Results:**

The monthly average number of new dementia cases registered in SveDem was 595, 415 and 470, respectively in the pre-COVID-19, COVID-19 and post-COVID-19 period. Compared to the pre-COVID-19 period, the monthly number of registrations decreased, but provision of the basic diagnostic work-up, its individual tests, and the use of cholinesterase inhibitors, memantine and antipsychotics were not significantly different in the COVID-19 period. Compared to the pre-COVID-19 period, new dementia diagnoses continued to be low in the post-COVID-19 period, but diagnosed individuals were more likely to receive the complete basic diagnostic work-up (OR 1.14, 95% CI 1.00–1.29), blood analysis (OR 1.88, 95% CI 1.44–2.49), computed tomography and magnetic resonance imaging (OR 1.22, 95% CI 1.01–1.48), occupational therapy assessment (OR 1.13, 95% CI 1.04–1.22), and memantine (OR 1.19, 95% CI 1.07–1.31).

**Conclusion:**

The quantity of new dementia registrations in SveDem decreased in the COVID-19 period and has not returned to pre-COVID-19 levels, but the quality of the work-ups which were conducted and registered in SveDem was similar or higher than in the pre-COVID-19 period. It is imperative to implement policies to increase SveDem registration with the aim of matching or exceeding pre-COVID-19 levels.

**Electronic Supplementary Material:**

Supplementary material is available in the online version of this article at 10.14283/jpad.2024.150.

## Introduction

The World Health Organization emphasizes the importance of timely access to dementia diagnosis, treatment and care in their “Global action plan on the public health response to dementia” (1). In Sweden, the National Board of Health and Welfare established quality indicators of dementia care, regarding goals for dementia diagnostics, including defining the complete basic diagnostic work-up and its individual tests, and targets for drug utilization (prescription of cholinesterase inhibitors, memantine and antipsychotics) (2). The COVID-19 pandemic disrupted care trajectories for specific diseases (3–11), and general health care services, such as physician consultations, specialist referrals and hospital admissions (7, 12). Previous studies also showed that the COVID-19 pandemic caused a reduction in the new diagnoses of diabetes, dementia, mental illness, or stroke (7–11). In Sweden, the Corona Commission reported that Sweden’s strategies against COVID-19 had failed to protect older adults and people in high risk groups (13). A recent study in Sweden also showed that the COVID-19 pandemic led to a decrease in new dementia diagnoses (14).

Dementia was underdiagnosed even before the COVID-19 pandemic (15). Previous studies observed that being an immigrant, living alone or having lower socioeconomic status predicted lower likelihood of receiving dementia diagnostics and treatment (16–18). The Swedish Registry for Cognitive/Dementia disorders – SveDem aims to register all new dementia diagnoses in Sweden. SveDem reports the number of new dementia diagnoses, quality of diagnostics and treatment annually, and evaluates how they meet national targets as quality indicators. It is imperative to investigate how the COVID-19 pandemic has influenced the quantity and quality of new dementia registrations, as well as the performance of dementia diagnostics and treatment.

The aim of this study is to explore whether the quantity of new registrations and the quality of dementia diagnostics and treatment differed in the pre-COVID-19, COVID-19 and post-COVID-19 periods. We hypothesized that compared to the pre-COVID-19 period, the performance of dementia diagnostics and treatment decreased in the COVID-19 period, and then recovered in the post-COVID-19 period.

## Methods

This study was reported according to the REporting of studies Conducted using Observational Routinely collected health Data (RECORD) statement (19) (Supplementary Table 1). The study was approved by the Swedish Ethical Review Authority (Decision number 2021-06246-01).

### Study Design and Setting

This was an observational study, based on the linkage of Swedish nationwide registers, including the Total Population Register, the Longitudinal Integrated Database for Health Insurance and Labor Market Studies - LISA, the National Patient Register, the Prescribed Drug Register, the Cause of Death Register, and SveDem. Descriptions of these registers were summarized in Supplementary Table 2. The linkage of these registers was performed using Swedish personal identification number by the National Board of Health and Welfare. Personal identification was pseudonymized before delivering to the researchers.

### Study Cohort

This study included people diagnosed with dementia and registered in SveDem. In Sweden, dementia is clinically diagnosed according to the 10th revision of the International Classification of Diseases (20), with separate criteria used for some specific types of dementia. The diagnostic process should follow the national guidelines established by the Swedish Board of Health and Welfare. According to the guidelines, the first level of dementia diagnosis is the basic diagnostic work-up (21). This basic work-up includes the completion of four tests: clock test, blood analysis, Mini-Mental State Examination (MMSE) and computed tomography or magnetic resonance imaging (CT-MRI), which are recorded in SveDem. This basic work-up also includes a patient interview, physical, functional and mental examination and interview with a person close to the patient, but these are not registered as variables in SveDem. If this leads to a diagnosis no further work-up is needed. Otherwise, the patient can be referred for additional examinations, such as neuropsychological assessment, usually through referral to a specialized clinic. For this study, the following inclusion criteria were used: (1) diagnosed with dementia between 01 January 2019 and 31 August 2021, (2) aged 45 years old or above at the date of dementia diagnosis, (3) alive at the time of their dementia diagnosis since some people die before their dementia diagnoses were registered in SveDem. A total of 16,245 people with dementia were retained for final analysis.

### Exposure

The main exposure, which was the time of dementia diagnosis registered in SveDem, included three categories: pre-COVID-19, COVID-19, and post-COVID-19 periods. According to the Swedish Corona Commission and the Public Health Agency, the COVID-19 pandemic in Sweden started from 01 March 2020 23. The pre-COVID-19 period was between 01 January 2019 and 29 February 2020. The COVID-19 period lasted from 01 March 2020 to 31 December 2020. The post-COVID-19 period was defined as the start of COVID-19 vaccination in Sweden, which ranged from 01 January 2021 until the latest available date of our dataset (31 August 2021).

### Outcome variables

Outcomes included dementia diagnostics and treatment, which were extracted from SveDem and the Swedish Prescribed Drug Register. The basic dementia diagnostic work-up and its four individual tests are important quality indicators in SveDem. We also examined additional dementia diagnostic tests, such as lumbar puncture, neuropsychological assessment, and occupational therapy assessment. Anti-dementia drugs were divided into cholinesterase inhibitors (donepezil, galantamine and rivastigmine) and memantine. The use of antipsychotics among persons with dementia was also investigated in this study. The prescription of drugs was examined at one, three and six months after the date of dementia diagnosis.

### Covariates

Covariates, which were extracted from the Total Population Register, LISA, and SveDem, included sociodemographic information such as age at dementia diagnosis, sex, living areas (urban, intermedia, rural), cohabitation status (living alone vs. cohabiting), education and annual individual income. The highest educational attainment before dementia diagnosis were categorized into compulsory education (years 1–9), upper secondary (years 10–12) and university (including college, university, master, or doctoral education). Individual income was defined as the total income that an individual obtained after paying taxes (including all types of income, allowances, or pension). Income was inflated into 2022 values with inflation rate from the Swedish Consumer Price Index (23), and then trichotomized into three equal parts. Comorbidities before dementia diagnosis were extracted from the National Patient Register, and then converted into the Charlson Comorbidity Index (24, 25). Another covariate extracted from SveDem was the type of diagnostic unit (primary care centers vs. specialized memory clinics) and living arrangements at dementia diagnosis (nursing homes vs. community dwellings). Types of dementia diagnosis, captured from SveDem, included Alzheimer’s disease, mixed dementia, vascular dementia, dementia with Lewy bodies, Parkinson disease’s dementia, frontotemporal dementia, unspecified dementia, and other dementias.

### Statistical analysis

Binary logistic regression was applied to examine the differences in dementia diagnostics and treatment between the pre-COVID-19 period with the COVID-19 or post-COVID-19 periods. For each outcome, two models of regression were performed to examine the associations with the time periods, and the robustness of the results. The first model was controlled for age at the date of dementia diagnosis, sex, and type of diagnostic unit. The second model was additionally controlled for living areas, cohabitation status, living arrangements, education, individual income, Charlson Comorbidity Index, and types of dementia diagnosis. Analysis on the use of cholinesterase inhibitors or memantine was only performed on persons with Alzheimer’s disease and mixed dementia since these have indication for treatment. Sub-group analysis was performed based on types of dementia diagnostic unit (primary care centers vs. specialized memory clinics).

Results of logistic regression were presented as odds ratio (OR) and 95% confidence interval (95% CI). All statistical tests were two tailed with a p-value less than 0.05 considered statistically significant. STATA version (16) (copyright StataCorp LLC, College Station, Texas, USA) and R 4.2.1 (The R Project for Statistical Computing) were used to perform the statistical analyses. After checking for missing values, a complete case analysis was applied.

## Results

### Description of the study cohort and the number of new dementia diagnoses

**Figure 1:**
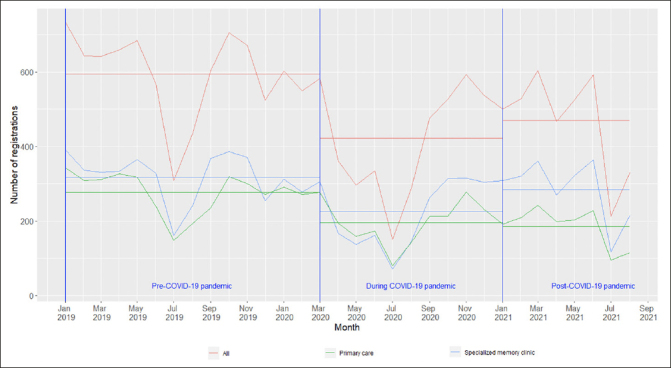
The number of dementia diagnosis in the pre-COVID-19, COVID-19 and post-COVID-19 periods

The monthly average number of new dementia cases declined: 595 cases in the pre-COVID-19 period, 415 cases in the COVID-19 period, and 470 cases in the post-COVID-19 period (Table 1). This downward trend of new dementia diagnosis happened in both primary care centers and specialized memory clinics in the COVID-19 period and persisted in the post-COVID-19 period (Figure 1). In each period, the mean ± standard deviation age at dementia diagnosis was 79.3 ± 7.9, 78.9 ± 7.9 and 78.9 ± 8.0 years old, respectively. In all three periods, women accounted for larger proportion of the sample, over 55%. More than 90% of people with dementia were living in community dwellings and over 50% of them were living alone at the time of dementia diagnosis in all periods.

**Table 1:** Characteristics of people with dementia diagnosed between January 2019 and August 2021

	**the pre-COVID-19 period (n = 8335)**	**the COVID-19 period (n = 4149)**	**p-value (vs. the pre-COVID-19 period)**	**the post-COVID-19 period (n = 3761)**	**p-value (vs. the pre-COVID-19 period)**
Age at dementia diagnosis, years, mean ± SD	79.3 ± 7.9	78.9 ± 7.9	0.006	78.9 ± 8.0	0.032
Female sex, n (%)	4698 (56.4)	2338 (56.4)	0.99	2075 (55.2)	0.22
Living arrangements, n (%)					
Community dwellings	7909 (95.0)	3967 (95.6)	0.10	3578 (95.2)	0.57
Nursing homes	419 (5.0)	181 (4.4)	0.10	180 (4.8)	0.57
Living areas, n (%)					
Urban	2752 (33.1)	1229 (29.6)	<0.001	1416 (37.7)	<0.001
Intermediate	2770 (33.3)	1473 (35.5)	0.012	1165 (31.0)	0.014
Rural	2802 (33.7)	1444 (34.8)	0.20	1176 (31.3)	0.011
Cohabitation status, n (%)					
Cohabiting	3909 (47.0)	2059 (49.7)	0.004	1789 (47.6)	0.50
Living alone	4415 (53.0)	2087 (50.3)	0.004	1968 (52.4)	0.50
Education, n (%)					
University/College	1148 (14.0)	621 (15.1)	0.082	571 (15.4)	0.045
Secondary education	4048 (49.2)	2115 (51.5)	0.016	1965 (52.8)	<0.001
Compulsory education	3025 (36.8)	1368 (33.3)	<0.001	1183 (31.8)	<0.001
Annually individual income, n (%)					
<173,158 SEK	2933 (35.2)	1412 (34.1)	0.19	1065 (28.3)	<0.001
Between 173,158 SEK and 219,968 SEK	2813 (33.8)	1342 (32.4)	0.11	1254 (33.4)	0.65
> 219,968 SEK	2578 (31.0)	1392 (33.6)	0.003	1438 (38.3)	<0.001
Charlson Comorbidity Index, n (%)					
0	3163 (37.9)	1585 (38.2)	0.78	1425 (37.9)	0.95
1	1328 (15.9)	651 (15.7)	0.73	601 (16.0)	0.95
2	1577 (18.9)	750 (18.1)	0.25	695 (18.5)	0.57
≥3	2267 (27.2)	1163 (28.0)	0.33	1040 (27.7)	0.60

The pre-COVID-19 period was between 01 January 2019 and 29 February 2020; The COVID-19 period was between 01 March 2020 and 31 December 2020; The post-COVID-19 period was between 01 January 2021 and 31 August 2021.

### Dementia diagnostics and treatment between the pre-COVID-19 and COVID-19 periods

Dementia diagnostics were performed on similar proportions of patients in the COVID-19 period, compared to the pre-COVID-19 period: basic diagnostic work-up (86.1% vs. 86.6%, p-value = 0.38), clock test (94.4% vs. 94.7%, p-value = 0.52), blood analysis (96.3% vs. 96.7%, p-value = 0.17), CT-MRI (93.5% vs. 93.8%, p-value = 0.56) and MMSE (97.6% vs. 98.0%, p-value = 0.26) (Table 2 and Supplementary Figures 1–11).

**Table 2:** Diagnostics and treatment for people with dementia diagnosed between January 2019 and August 2021

	**the pre-COVID-19 period (n = 8335)**	**the COVID-19 period (n = 4149)**	**p-value (vs. the pre-COVID-19 period)**	**the post-COVID-19 period (n = 3761)**	**p-value (vs. the pre-COVID-19 period)**
Diagnostic unit, n (%)					
Specialized memory clinics	4457 (53.5)	2186 (52.7)	0.41	2279 (60.6)	<0.001
Primary care centers	3878 (46.5)	1963 (47.3)	0.41	1482 (39.4)	<0.001
Type of dementia diagnosis, n (%)					
Alzheimer’s disease	3081 (37.0)	1621 (39.1)	0.022	1456 (38.7)	0.066
Mixed dementia	1678 (20.1)	777 (18.7)	0.063	827 (22.0)	0.020
Vascular dementia	1626 (19.5)	705 (17.0)	<0.001	671 (17.8)	0.030
Dementia with Lewy bodies	198 (2.4)	130 (3.1)	0.013	109 (2.9)	0.091
Parkinson’s disease dementia	109 (1.3)	65 (1.6)	0.25	60 (1.6)	0.21
Frontotemporal dementia	152 (1.8)	88 (2.1)	0.25	87 (2.3)	0.073
Unspecified dementia	1281 (15.4)	664 (16.0)	0.36	508 (13.5)	0.008
Other dementias	210 (2.5)	99 (2.4)	0.65	43 (1.1)	<0.001
Dementia diagnostic examinations, n (%)					
The basic diagnostic work-up	7174 (86.1)	3595 (86.6)	0.38	3341 (88.8)	<0.001
Clock test	7870 (94.4)	3929 (94.7)	0.52	3549 (94.4)	0.90
Blood analysis	8023 (96.3)	4014 (96.7)	0.17	3694 (98.2)	<0.001
CT-MRI	7796 (93.5)	3892 (93.8)	0.56	3594 (95.6)	<0.001
Mini Mental State Examination - MMSE	8138 (97.6)	4064 (98.0)	0.26	3668 (97.5)	0.72
MMSE score, median (IQR)	22.0 (7.0)	22.0 (7.0)	0.34	22.0 (6.0)	0.045
Lumbar puncture	2285 (27.4)	1106 (26.7)	0.37	1139 (30.3)	0.001
Neuropsychological assessment	1453 (17.4)	646 (15.6)	0.009	714 (19.0)	0.039
Occupational therapy assessment	4590 (55.1)	2341 (56.4)	0.15	2217 (58.9)	<0.001
Drug utilization after dementia diagnosis, n (%)					
Cholinesterase inhibitors (1 month)	1031 (21.7)	537 (22.4)	0.48	501 (21.9)	0.79
Cholinesterase inhibitors (3 months)	1979 (41.6)	1007 (42.0)	0.74	937 (41.0)	0.67
Cholinesterase inhibitors (6 months)	2298 (48.3)	1191 (49.7)	0.27	1058 (46.3)	0.13
Memantine (1 month)	454 (9.5)	203 (8.5)	0.14	248 (10.9)	0.083
Memantine (3 months)	841 (17.7)	454 (18.9)	0.19	504 (22.1)	<0.001
Memantine (6 months)	1182 (24.8)	631 (26.3)	0.18	681 (29.8)	<0.001
Antipsychotics (1 month)	135 (1.6)	78 (1.9)	0.29	78 (2.1)	0.079
Antipsychotics (3 months)	310 (3.7)	174 (4.2)	0.20	158 (4.2)	0.20
Antipsychotics (6 months)	490 (5.9)	272 (6.6)	0.14	248 (6.6)	0.13

The pre-COVID-19 period was between 01 January 2019 and 29 February 2020. The COVID-19 period was between 01 March 2020 and 31 December 2020. The post-COVID-19 period was between 01 January 2021 and 31 August 2021. CT-MRI computed tomography or magnetic resonance imaging. IQR Inter Quartile Range. The use of cholinesterase inhibitors and memantine was calculated on people with Alzheimer’s disease and mixed dementia (the pre-COVID-19 period (n = 4759), the COVID-19 period (n = 2398), the post-COVID-19 period (n = 2283)).

**Figure 2:**
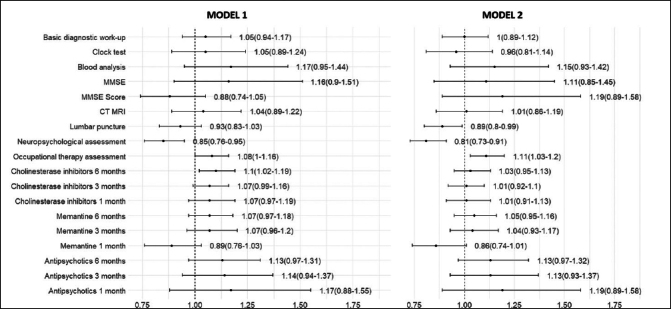
Odds ratio of dementia diagnostics and treatment between the COVID-19 and the pre-COVID-19 periods Model 1 was performed with binary logistic regression and controlled for age at the date of dementia diagnosis, sex, and type of diagnostic unit. Model 2 was additionally adjusted for living areas, cohabitation status, living arrangements, education, individual income, Charlson Comorbidity Index, and types of dementia diagnosis.

Fully adjusted regression models showed no significant association between the time of dementia diagnosis (the pre-COVID-19 versus. the COVID-19 periods) and the performance of basic diagnostic work-up and its individual tests, as well as the prescription of cholinesterase inhibitors, memantine and antipsychotics (Figure 2). However, in the COVID-19 period, the likelihood of getting additional diagnostic tests were significantly different: lumbar puncture (OR 0.89, 95% CI 0.80–0.99), neuropsychological assessment (OR 0.81, 95% CI 0.73–0.91), and occupational therapy assessment (OR 1.11, 95% CI 1.03–1.20).

### Dementia diagnostics and treatment between the pre-COVID-19 and post-COVID-19 periods

The performance of diagnostic tests and treatment was significantly higher in the post-COVID-19 compared to the pre-COVID-19 period (Table 2 and Supplementary Figures 1–11): the basic diagnostic work-up (88.8% vs. 86.1%, p-value < 0.001), blood analysis (98.2% vs. 96.3%, p-value < 0.001), CT-MRI (95.6% vs. 93.5%, p-value < 0.001) and memantine in three months (22.1% vs. 17.7%, p-value < 0.001) and six months (29.8% vs. 24.8%, p-value < 0.001) after dementia diagnosis. No significant difference was observed in the prescription of cholinesterase inhibitors and antipsychotics.

**Figure 3:**
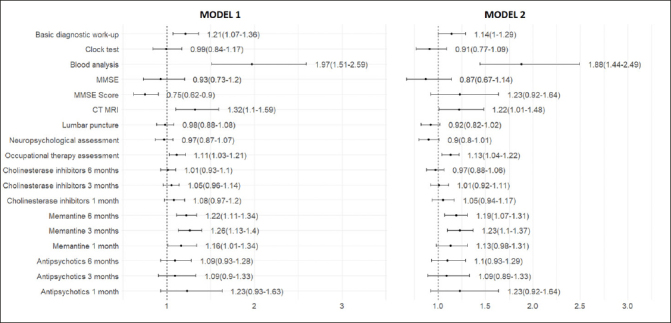
Odds ratio of dementia diagnostics and treatment between the post-COVID-19 and the pre-COVID-19 periods Model 1 was performed with binary logistic regression and controlled for age at the date of dementia diagnosis, sex, and type of diagnostic unit. Model 2 was additionally adjusted for living areas, cohabitation status, living arrangements, education, individual income, Charlson Comorbidity Index, and types of dementia diagnosis.

Fully adjusted regression models showed that in the post-COVID-19 period, persons with dementia were more likely to receive the basic diagnostic work-up (OR 1.14, 95% CI 1.00–1.29), blood analysis (OR 1.88, 95% CI 1.44–2.49), CT – MRI (OR 1.22, 95% CI 1.01–1.48), occupational therapy assessment (OR 1.13, 95% CI 1.04–1.22), and memantine at three months (OR 1.23, 95% CI 1.10–1.37) and six months (OR 1.19, 95% CI 1.07–1.31) after dementia diagnosis, compared to individuals in the pre-COVID-19 period (Figure 3).

### Subgroup analysis on diagnostic units

More persons with dementia were diagnosed in the specialized memory clinics throughout the three periods, except for a higher number of people diagnosed in the primary care centers between March and August 2020, which was commonly known as the first COVID-19 wave in Sweden (Figure 1). The proportion of persons with dementia diagnosed at specialized memory clinics in the pre-COVID-19 period (53.5%) was not significantly different from the COVID-19 period (52.7%), but significantly lower compared to that in the post-COVID-19 period (60.6%) (Table 2).

Compared to the pre-COVID-19 period, persons with dementia in the post-COVID-19 period had higher chances of undergoing the basic diagnostic work-up (OR 1.31, 95% CI 1.11–1.55) and blood analysis (OR 2.46, 95% CI 1.75–3.54) when diagnosed in primary care centers, but lower likelihood of receiving clock test (OR 0.75, 95% CI 0.59–0.94) and MMSE (OR 0.63, 95% CI 0.44–0.92) when diagnosed in specialized memory clinics (Supplementary Figures 12 and 13). Compared to the pre-COVID-19 period, the likelihood of using memantine at six months after dementia diagnosis was higher in the COVID-19 period (OR 1.16, 95% CI 1.02–1.31), and in the post-COVID-19 period (OR 1.28, 95% CI 1.14–1.44) among people diagnosed in the specialized memory clinics.

## Discussion

The results of our study showed a reduction in the quantity of dementia registrations in the COVID-19 period. This declining trend continued into the post-COVID-19 period and persisted up to the date of data extraction (31 August 2021). All changes in the quality of diagnosis and treatment must be interpreted within this context of loss of total number of registrations. In comparison to the pre-COVID-19 period, the chance of receiving the basic diagnostic work-up, blood analysis and CT-MRI for those who did receive a diagnosis was not significantly different in the COVID-19 period but was significantly higher in the post-COVID-19 period. Persons with dementia had higher likelihood of receiving additional tests, such as occupational therapy assessment both during and in the post-COVID-19 period. However, they had a lower chance of receiving lumbar puncture and neuropsychological assessment in the COVID-19 period. No significant difference up to six months after the diagnosis date was found in the prescription of cholinesterase inhibitors, memantine and antipsychotics, except for higher use of memantine at three and six months after dementia diagnosis in the post-COVID-19 period.

To our knowledge, this is the first study exploring the changes in the provision of dementia diagnostic tests and the prescription of drugs for persons with dementia in the pre-COVID-19, COVID-19 and post-COVID-19 periods. In this nationwide cohort study, we observed that the total number of new diagnoses decreased, but the performance of dementia diagnostics and treatment for those patients who were diagnosed and registered in SveDem was not significantly different in the COVID-19 period, compared to the pre-COVID-19 period, apart from lower chances of receiving lumbar puncture and neuropsychological assessment, or higher chances of receiving occupational therapy assessment. Our findings arrive in the context of previous studies which showed a reduction in the supply of general health care services (7, 11, 12), or specific care services for persons having diabetes, mental illness, stroke and so on (3–11). Divergent methodology and population as well as difference in the definition of COVID-19 and post-COVID-19 periods probably cause the difference in results between our study and other studies.

Recent studies in Sweden and the USA reported a substantial decline in dementia diagnosis incidence because of COVID-19 (8, 14). In our study, we observed this downward trend of new dementia diagnosis both in specialized memory clinics and primary care in the COVID-19 period, compared to the pre-COVID-19 period. This decline might be attributed to disruptions in healthcare services, alterations in patient behaviour, and diminished access to dementia diagnostics. The crisis of health care system in the COVID-19 period led to the postponement or cancellation of non-urgent medical appointments and procedures, including those for dementia diagnostics. Furthermore, patients and their caregivers altered their behaviour in the COVID-19 period. The fear of contracting COVID-19 deterred some patients from seeking health care services, including evaluations for dementia. Additionally, recommendations on social distancing and restrictions on in-person interactions especially for elderly may have reduced the chances for family members and caregivers to observe and report changes in cognitive function or behaviour, potentially leading to a delayed diagnosis. Consequently, more advanced stages of dementia might occur in these patients, as shown by the increased proportion of memantine usage in the post-COVID-19 period. Patients come to memory clinics through referrals from primary care. Neuropsychology is rarely available to primary care in Sweden: the shift towards primary care diagnoses, and the subsequent reduction in neuropsychological testing, could have led to fewer dementia diagnoses overall or lower diagnostic precision, but the latter was not assessed by our study. The higher likelihood of receiving occupational therapy assessment during and after the COVID-19 periods is a consequence of this shift to primary care, since this type of testing is available in primary care, whereas neuropsychology is not. However, the performance of the basic diagnostic work-up which is also conducted in person remained high: our interpretation is that this part of the work-up, which also serves to identify and exclude treatable causes of cognitive decline, was prioritized.

Our results indicate that the quality of diagnostic work-ups remained high, despite the drop in quantity. It is possible that the reduction in number of diagnosed patients led to more resources and better quality of care for those who did receive a diagnosis. Compared to the pre-COVID-19 period, the odds of receiving the basic diagnostic work-up, blood analysis, and CT-MRI was not significantly different in the COVID-19 period but was significantly higher in the post-COVID-19 period. In the first wave of COVID-19 in Sweden (roughly between March and August 2020), many specialized memory clinics closed so that medical personnel could work with hospitalized patients instead and dementia diagnosis fell on primary care. The reallocation from specialized memory clinics to primary care centers of dementia diagnostics in the COVID-19 period in Sweden was also shown in a recent study (15). This shift to primary care was achieved while maintaining compliance with the national guidelines.

The higher likelihood of receiving occupational therapy assessment during and after the COVID-19 period is a consequence of this shift to primary care, since this type of testing is often available in primary care in Sweden, whereas neuropsychological testing is not.

### Limitations

There were several limitations in our study. First, the coverage of SveDem ranged between 23% and 29%, depending on calculation on the estimated incidence of dementia (26). Moreover, it was impossible to estimate the exact number of persons with dementia because not everyone with dementia can be identified or is ever diagnosed. Despite its imperfect coverage, the national nature of SveDem contributes to its representativeness. This study aimed to describe the quantity and quality of dementia diagnostics and treatment during the different COVID-19 periods. The causes behind these trends are multifactorial and are beyond the scope of this article. The use of telemedicine, which was more common in the COVID-19 period, was not available as a variable in the registries. Thus, it is impossible for us to evaluate the impact of telemedicine on the dementia diagnostics and treatment in the COVID-19 period.

The strength of this study was the combination of nationwide registers, enabling the large sample size and the generalizability of the study. Another advantage was a large cohort of persons with dementia from SveDem, which is the largest clinical quality registry of dementia in the world. This is the first study that explored how the supply of dementia diagnostics and treatment changed during and after the COVID-19 pandemic. This topic is important for the assurance of quality of care for persons with dementia and for the preparedness of health care systems toward the next pandemics.

## Conclusion

The number of new dementia diagnoses dropped in the COVID-19 period and in the post-COVID-19 period, but the quality of the diagnoses remained similar. Compared to the pre-COVID-19 period, the performance of the basic diagnostic work-up and its individual tests, and the prescription of cholinesterase inhibitors, memantine and antipsychotics were not significantly different in the COVID-19 period. In the post-COVID-19 period, persons with dementia had higher likelihood of receiving the basic diagnostic work-up, blood analysis, neuroimaging and memantine. Further studies should be conducted to explore the impact of policies against COVID-19 on the quality of care for persons with dementia. Policies to increase registration in SveDem and counteract pandemic and post-pandemic losses in registrations should be created and implemented.

## SUPPLEMENTARY MATERIAL

Supplementary material, approximately 1.24 MB.

## Data Availability

*Data availability statement:* No data are available. The entities responsible for the original data and the Swedish law do not allow for sharing of the data from the Swedish national registers.

## Notes

*Ethical standard statement:* Ethical approval was given by the Swedish Ethical Review Authority (Decision number 2021-06246-01). This research was conducted following the Helsinki Declaration.
