# Supplementary material for: Effects of the COVID-19 Pandemic on the Number of New Dementia Diagnoses and the Quality of Dementia Diagnostics and Treatment
Source: J Prev Alzheimers Dis. 2024 Aug 10;11(6):1703–11. doi: 10.14283/jpad.2024.150 (PMC11573820; doi:10.14283/jpad.2024.150)
Supplement: Supplementary file 1 — Supplementary material, approximately 1.24 MB. [file 42414_2024_150_MOESM1_ESM.docx]

**Effects of the COVID-19 pandemic on the number of new dementia diagnoses and the quality of dementia diagnostics and treatment**

**SUPPLEMENTARY MATERIAL**

**Supplementary materials**

[Supplementary Table 1. The RECORD statement – checklist of items, extended from the STROBE statement, that should be reported in observational studies using routinely collected health data 1](#_Toc170220212)

[Supplementary Table 2. Data source of the study 3](#_Toc170220213)

[Supplementary Figure 1. The performance of the basic diagnostic work-up in the pre-COVID-19, COVID-19 and post-COVID-19 periods 4](#_Toc170220214)

[Supplementary Figure 2. The performance of clock test in the pre-COVID-19, COVID-19 and post-COVID-19 periods 5](#_Toc170220215)

[Supplementary Figure 3. The performance of blood analysis in the pre-COVID-19, COVID-19 and post-COVID-19 periods 6](#_Toc170220216)

[Supplementary Figure 4. The performance of CT-MRI in the pre-COVID-19, COVID-19 and post-COVID-19 periods 7](#_Toc170220217)

[Supplementary Figure 5. The performance of Mini-Mental State Examination in the pre-COVID-19, COVID-19 and post-COVID-19 periods 8](#_Toc170220218)

[Supplementary Figure 6. The performance of lumbar puncture in the pre-COVID-19, COVID-19 and post-COVID-19 periods 9](#_Toc170220219)

[Supplementary Figure 7. The performance of neuropsychological assessment in the pre-COVID-19, COVID-19 and post-COVID-19 periods 10](#_Toc170220220)

[Supplementary Figure 8. The performance of occupational therapy assessment in the pre-COVID-19, COVID-19 and post-COVID-19 periods 11](#_Toc170220221)

[Supplementary Figure 9. The prescription of cholinesterase inhibitors in the pre-COVID-19, COVID-19 and post-COVID-19 periods 12](#_Toc170220222)

[Supplementary Figure 10. The prescription of memantine in the pre-COVID-19, COVID-19 and post-COVID-19 periods 13](#_Toc170220223)

[Supplementary Figure 11. The prescription of antipsychotics in the pre-COVID-19, COVID-19 and post-COVID-19 periods 14](#_Toc170220224)

[Supplementary Figure 12. Odds ratio of dementia diagnostics and treatment between the COVID-19 and pre-COVID-19 periods. 15](#_Toc170220225)

[Supplementary Figure 13. Odds ratio of dementia diagnostics and treatment between the post-COVID-19 and pre-COVID-19 periods 16](#_Toc170220226)

[References for Supplementary Material 17](#_Toc170220227)

## Supplementary Table 1. The RECORD statement – checklist of items, extended from the STROBE statement, that should be reported in observational studies using routinely collected health data (1)

| **RECORD items** | | **Details** | **Location** |
| --- | --- | --- | --- |
| **Title and abstract** | 1 | (1) The type of data used should be specified in the title or abstract. When possible, the name of the databases used should be included. | Page 2 |
|  |  | (2) If applicable, the geographic region and timeframe within which the study took place should be reported in the title or abstract. | Page 2 |
|  |  | (3) If linkage between databases was conducted for the study, this should be clearly stated in the title or abstract. | Page 2 |
| **Introduction** | | |  |
| Background/rationale | 2 | Explain the scientific background and rationale for the investigation being reported | Page 4 |
| Objectives | 3 | State specific objectives, including any prespecified hypotheses | Pages 4-5 |
| **Methods** | | |  |
| Study design | 4 | Present key elements of study design early in the paper | Page 6 |
| Setting | 5 | Describe the setting, locations, and relevant dates, including periods of recruitment, exposure, follow-up, and data collection | Page 6 |
| Participants | 6 | (*1*) The methods of study population selection (such as codes or algorithms used to identify subjects) should be listed in detail. If this is not possible, an explanation should be provided. | Page 6 |
|  |  | (*2*) Any validation studies of the codes or algorithms used to select the population should be referenced. If validation was conducted for this study and not published elsewhere, detailed methods and results should be provided. | Not applicable |
|  |  | (3) If the study involved linkage of databases, consider use of a flow diagram or other graphical display to demonstrate the data linkage process, including the number of individuals with linked data at each stage. | Not applicable |
| Variables | 7 | A complete list of codes and algorithms used to classify exposures, outcomes, confounders, and effect modifiers should be provided. If these cannot be reported, an explanation should be provided. | Pages 6-8 |
| Data sources/ measurement | 8* | For each variable of interest, give sources of data and details of methods of assessment (measurement). Describe comparability of assessment methods if there is more than one group | Pages 6-8 |
| Bias | 9 | Describe any efforts to address potential sources of bias | Page 13 |
| Study size | 10 | Explain how the study size was arrived at | Page 6 |
| Quantitative variables | 11 | Explain how quantitative variables were handled in the analyses. If applicable, describe which groupings were chosen and why | Page 8 |
| Statistical methods | 12 | (*a*) Describe all statistical methods, including those used to control for confounding | Page 8 |
|  |  | (*b*) Describe any methods used to examine subgroups and interactions | Page 8 |
|  |  | (*c*) Explain how missing data were addressed | Page 8 |
|  |  | (*d*) If applicable, explain how loss to follow-up was addressed | Not applicable |
|  |  | (*e*) Describe any sensitivity analyses | Page 8 |
| Data access and cleaning methods |  | Authors should describe the extent to which the investigators had access to the database population used to create the study population. | Page 6 |
|  |  | Authors should provide information on the data cleaning methods used in the study. | Page 6 |
|  |  | State whether the study included person-level, institutional-level, or other data linkage across two or more databases. The methods of linkage and methods of linkage quality evaluation should be provided. | Page 6 |
| **Results** | | |  |
| Participants | 13* | (a) Describe in detail the selection of the persons included in the study (i.e., study population selection) including filtering based on data quality, data availability and linkage. The selection of included persons can be described in the text and/or by means of the study flow diagram. | Page 6 |
|  |  | (b) Give reasons for non-participation at each stage | Page 6 |
|  |  | (c) Consider use of a flow diagram | Not applicable |
| Descriptive data | 14* | (a) Give characteristics of study participants (eg demographic, clinical, social) and information on exposures and potential confounders | Page 9 |
|  |  | (b) Indicate number of participants with missing data for each variable of interest | Page 9 |
|  |  | (c) Summarise follow-up time (eg, average and total amount) | Page 9 |
| Outcome data | 15* | Report numbers of outcome events or summary measures over time | Pages 9-10; Tables 1-2 |
| Main results | 16 | (*a*) Give unadjusted estimates and, if applicable, confounder-adjusted estimates and their precision (eg, 95% confidence interval). Make clear which confounders were adjusted for and why they were included | Figures 2-3 |
|  |  | (*b*) Report category boundaries when continuous variables were categorized | Not applicable |
|  |  | (*c*) If relevant, consider translating estimates of relative risk into absolute risk for a meaningful time period | Not applicable |
| Other analyses | 17 | Report other analyses done—eg analyses of subgroups and interactions, and sensitivity analyses | Supplementary Tables and Figures |
| **Discussion** | | |  |
| Key results | 18 | Summarise key results with reference to study objectives | Page 11 |
| Limitations | 19 | Discuss the implications of using data that were not created or collected to answer the specific research question(s). Include discussion of misclassification bias, unmeasured confounding, missing data, and changing eligibility over time, as they pertain to the study being reported. | Page 13 |
| Interpretation | 20 | Give a cautious overall interpretation of results considering objectives, limitations, multiplicity of analyses, results from similar studies, and other relevant evidence | Pages 11-12 |
| Generalisability | 21 | Discuss the generalisability (external validity) of the study results | Pages 11-12 |
| **Other information** | | |  |
| Funding | 22 | Give the source of funding and the role of the funders for the present study and, if applicable, for the original study on which the present article is based | Page 14 |
| Accessibility of protocol, raw data, and programming code |  | Authors should provide information on how to access any supplemental information such as the study protocol, raw data, or programming code. | Page 14 |

## Supplementary Table 2. Data source of the study

| **Register** | **Founded year** | **Characteristics** | **Externally validated** | **Coverage** | **Data extracted for our study** |
| --- | --- | --- | --- | --- | --- |
| The Total Population Register (2) | 1968 | The register provides complete population-based data, which consists of 100 % of births and deaths, 95 % of immigrations and 91 % of emigrations  Data are reported within 30 days and with a higher proportion over time. | Yes | Nationwide | Sex  Month and year of birth  Country of birth  Municipality of residence  Marital status  Date of immigration and emigration |
| The Longitudinal Integrated Database for Health Insurance and Labor Market Studies (LISA) (3) | 1990 | LISA is updated annually with a roughly 15-month delay.  LISA contains data of all individuals aged ≥ 16 years who are registered in Sweden as of December 31 each year regarding education, income, occupation and so on. | Yes | Nationwide | Highest educational attainment  Disposable individual income |
| The National Patient Register (4) | 1964 | The register collects data from 99% of all somatic and psychiatric hospital discharges, and over 80% of hospital-based outpatient care. | About 85-95% of all diagnoses are valid | Nationwide | Date of admission and discharge  Main diagnosis  Secondary diagnoses |
| The Prescribed Drug Register (5, 6) | 2005 | The register reports contains all dispensed prescription drugs in pharmacy in Sweden (equal to 85% of all sold defined daily doses)  The register does not cover the remaining 15%, including over-the-counter drugs and drugs administered in hospitals. | Yes | Nationwide | Date of prescription  Date of dispensing  ATC code |
| The Cause of Death Register (7) | 1952 | The register reports all deaths, with an underlying cause of death. | Yes | Nationwide | Cause of death  Date of death |
| The Swedish registry for cognitive/dementia disorders – SveDem (8) | 2007 | This clinical quality register of dementia contains information of persons with dementia at baseline and annual follow-up, regarding demographics, diagnostics, treatment, and care. | Yes | All persons with dementia diagnosed at memory clinic, 78% of primary care centers, and many nursing homes. | Clinically confirmed dementia cases. Dementia diagnostics and treatment. |

## Supplementary Figure 1. The performance of the basic diagnostic work-up in the pre-COVID-19, COVID-19 and post-COVID-19 periods


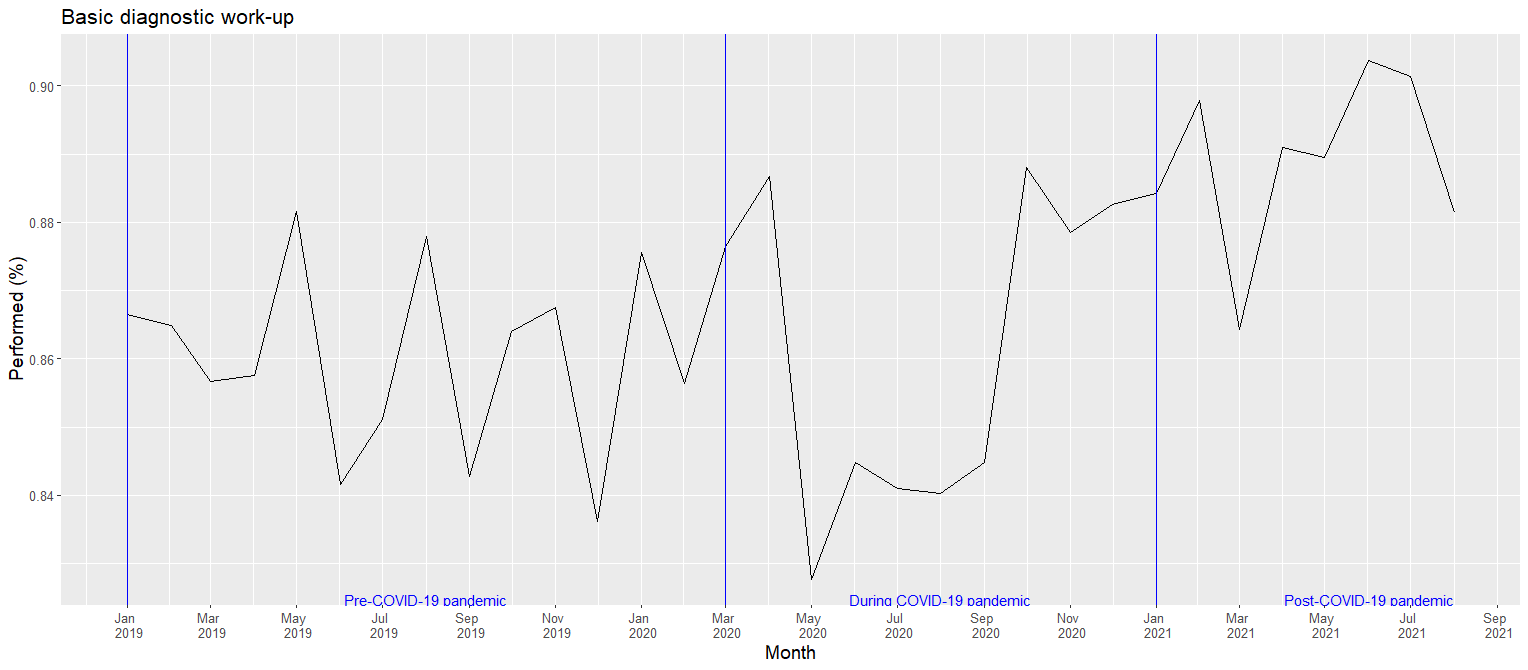


## Supplementary Figure 2. The performance of clock test in the pre-COVID-19, COVID-19 and post-COVID-19 periods


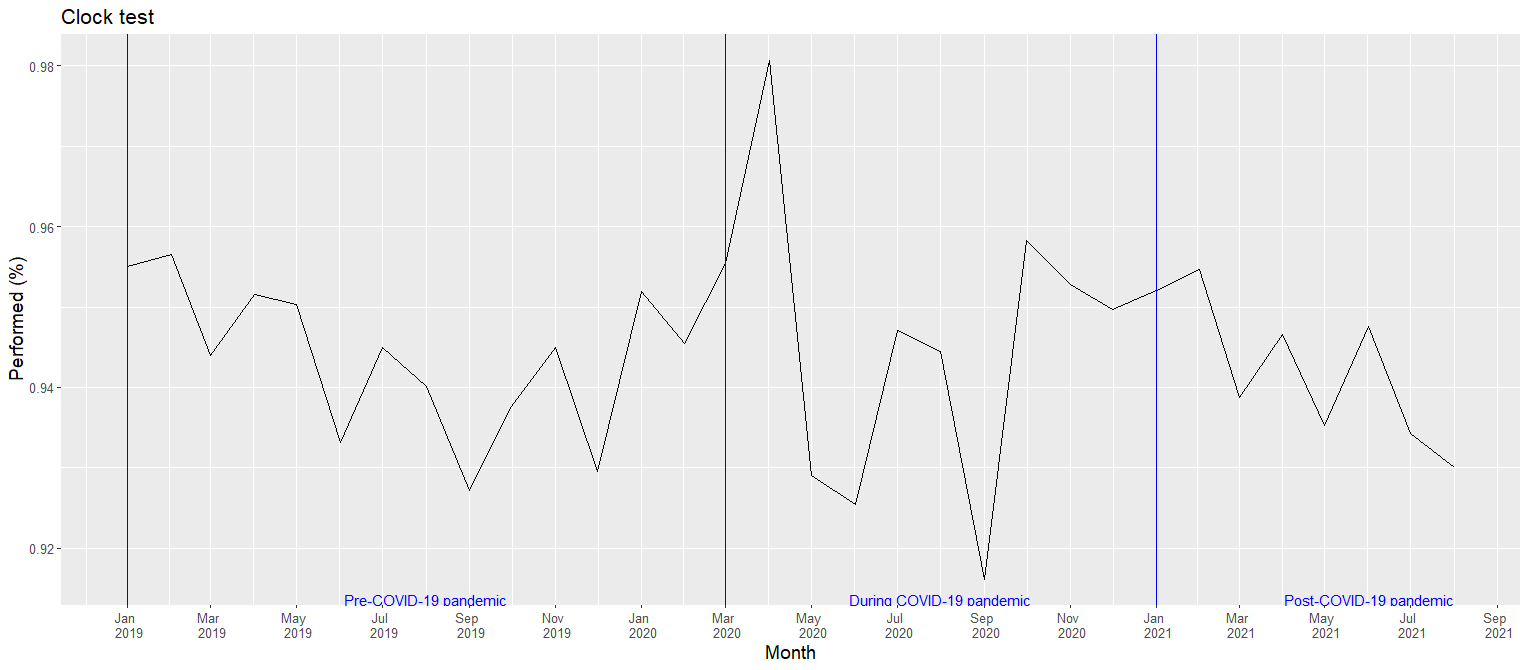


## Supplementary Figure 3. The performance of blood analysis in the pre-COVID-19, COVID-19 and post-COVID-19 periods


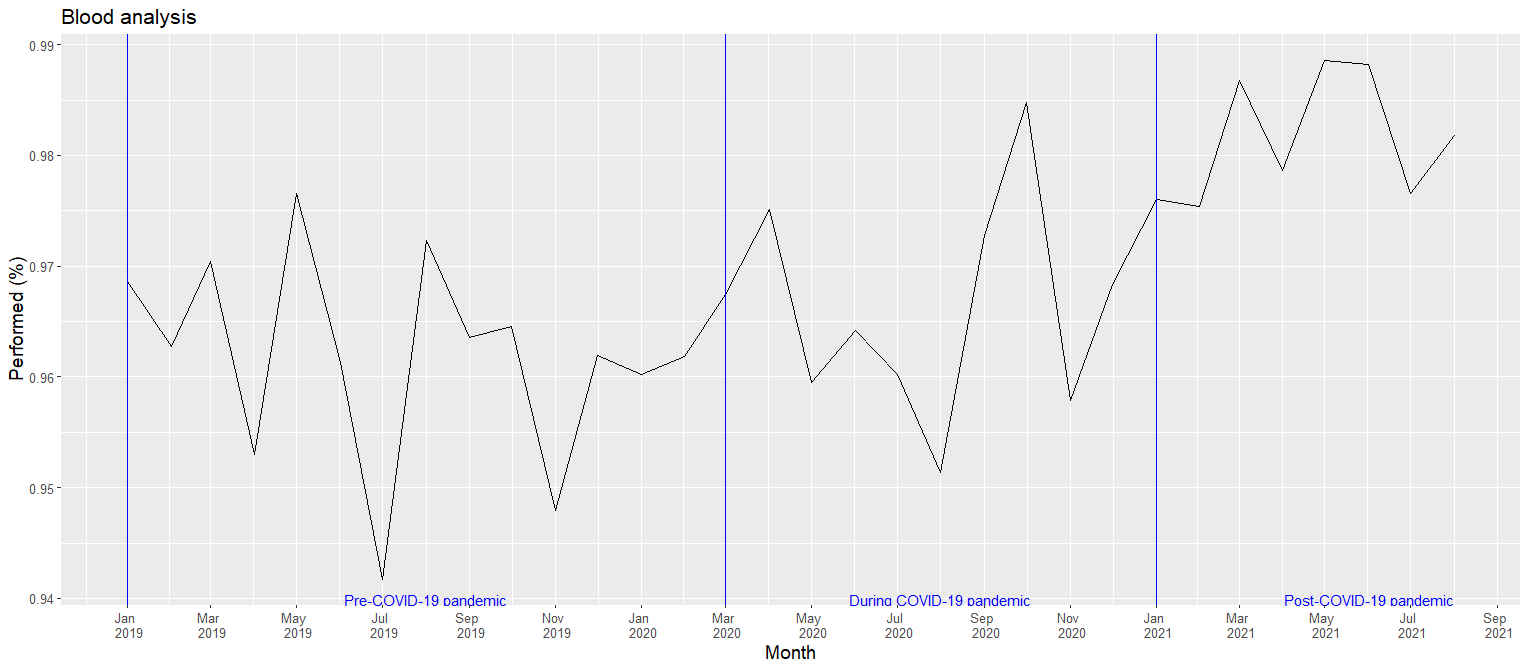


## Supplementary Figure 4. The performance of CT-MRI in the pre-COVID-19, COVID-19 and post-COVID-19 periods


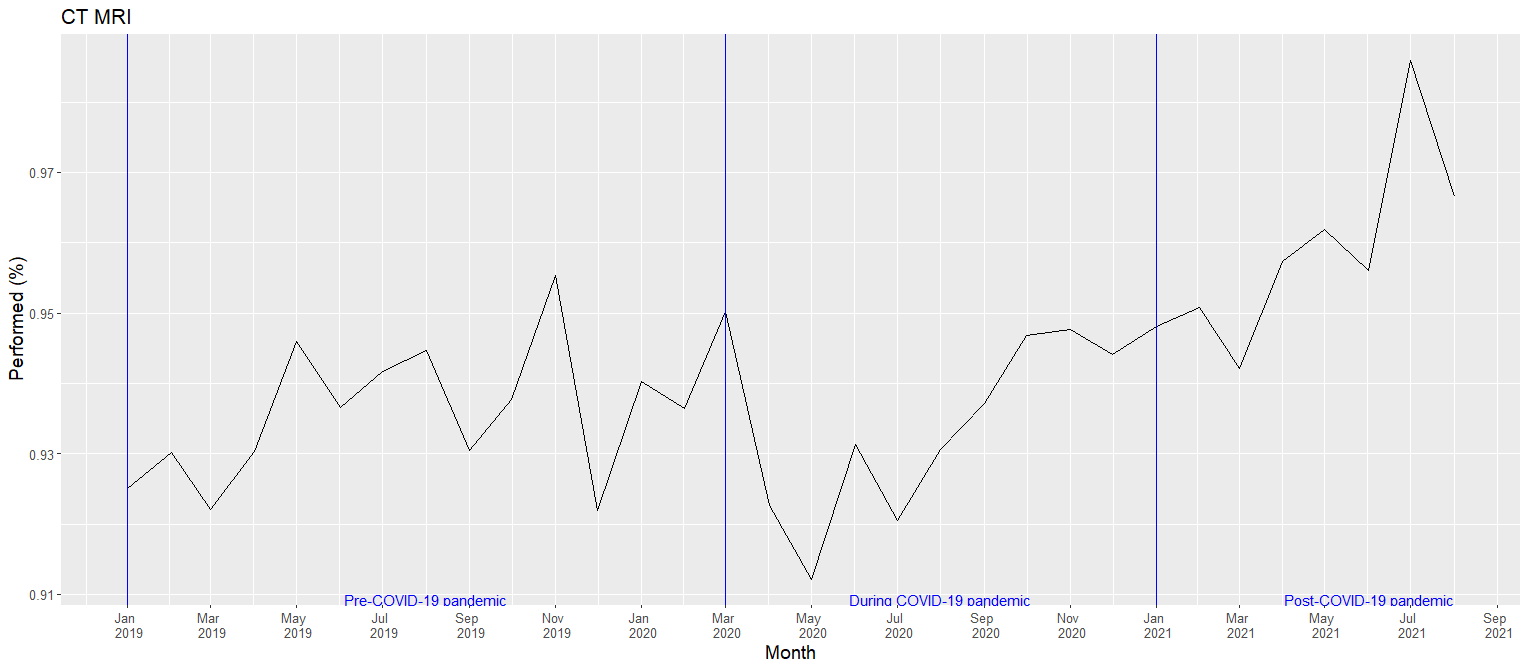


## Supplementary Figure 5. The performance of Mini-Mental State Examination in the pre-COVID-19, COVID-19 and post-COVID-19 periods


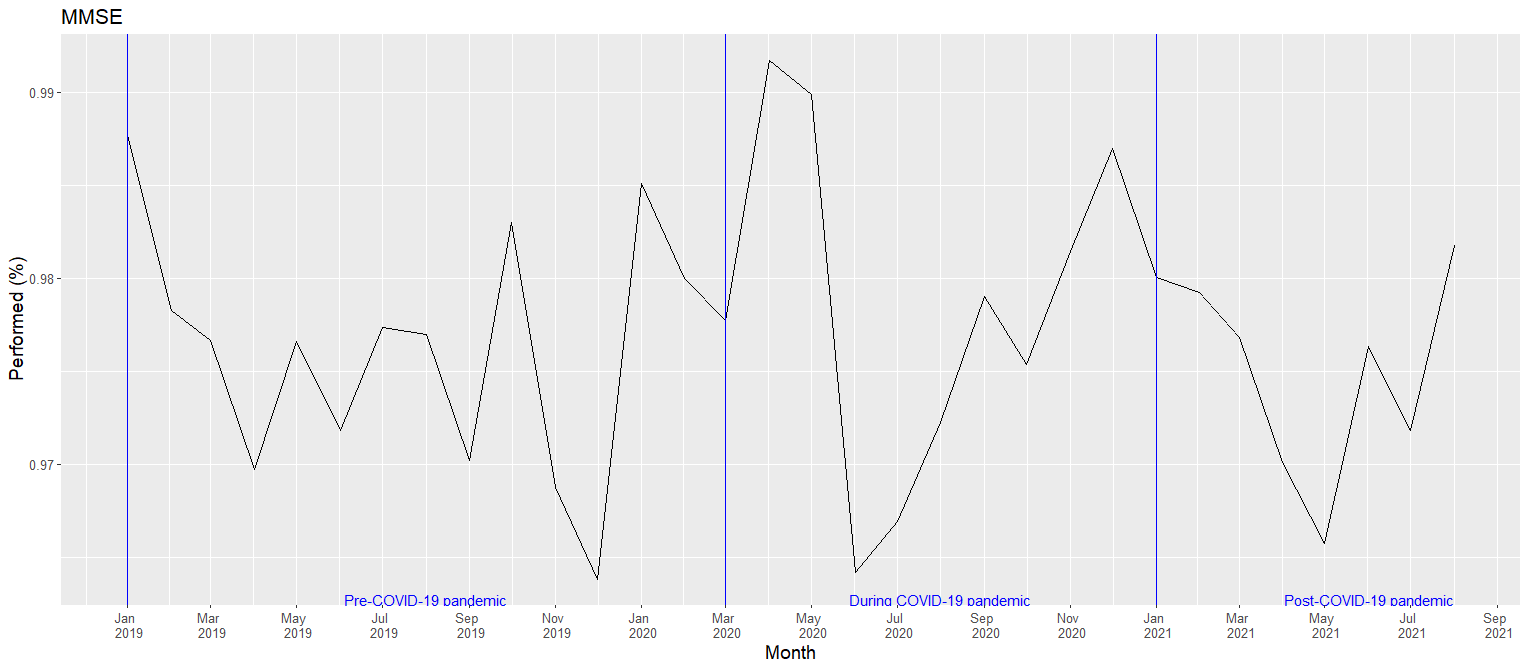


## Supplementary Figure 6. The performance of lumbar puncture in the pre-COVID-19, COVID-19 and post-COVID-19 periods


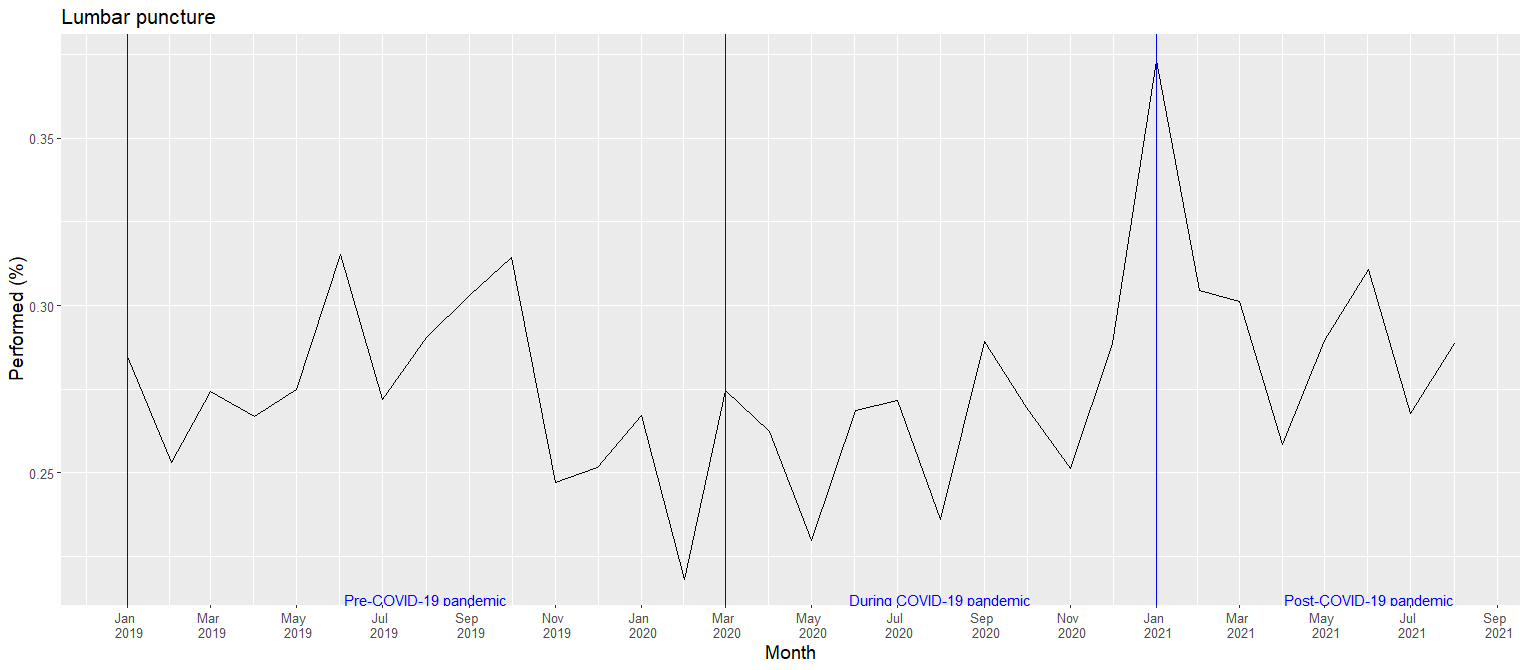


## Supplementary Figure 7. The performance of neuropsychological assessment in the pre-COVID-19, COVID-19 and post-COVID-19 periods

**
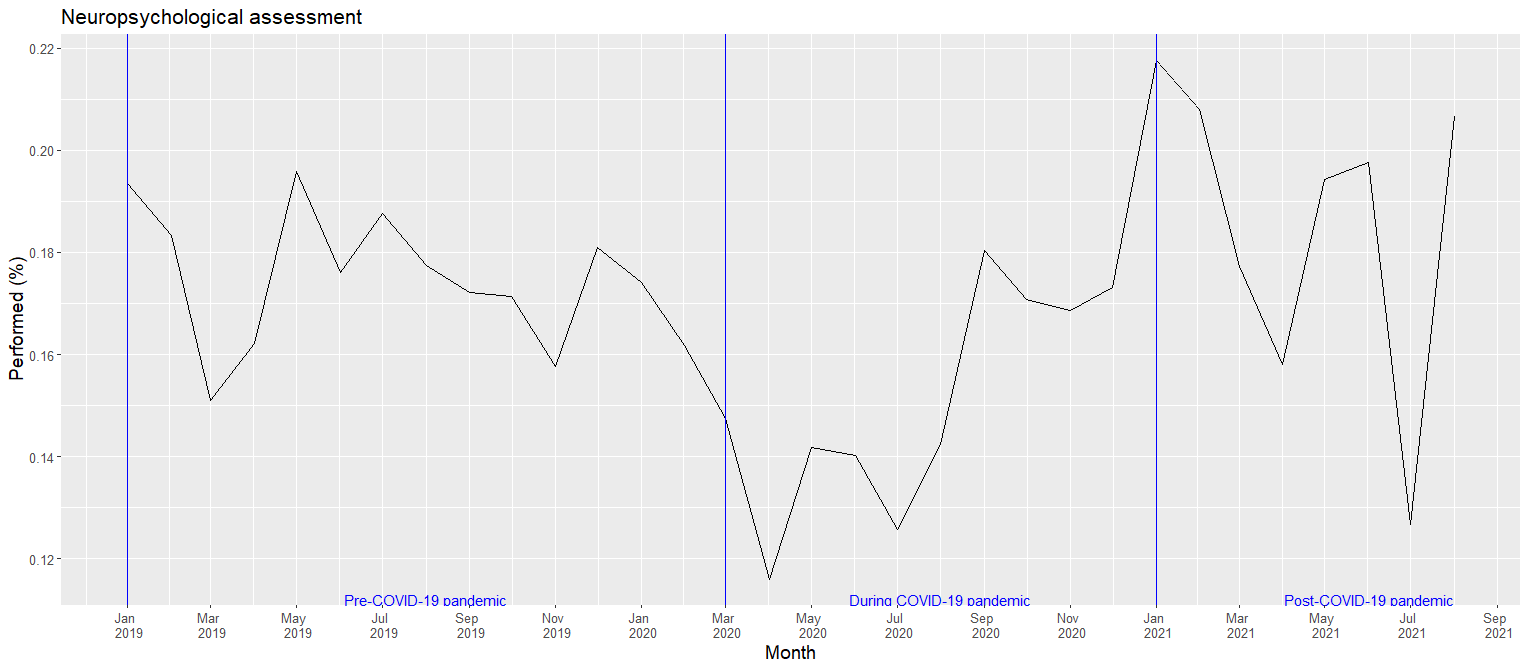
**

## Supplementary Figure 8. The performance of occupational therapy assessment in the pre-COVID-19, COVID-19 and post-COVID-19 periods


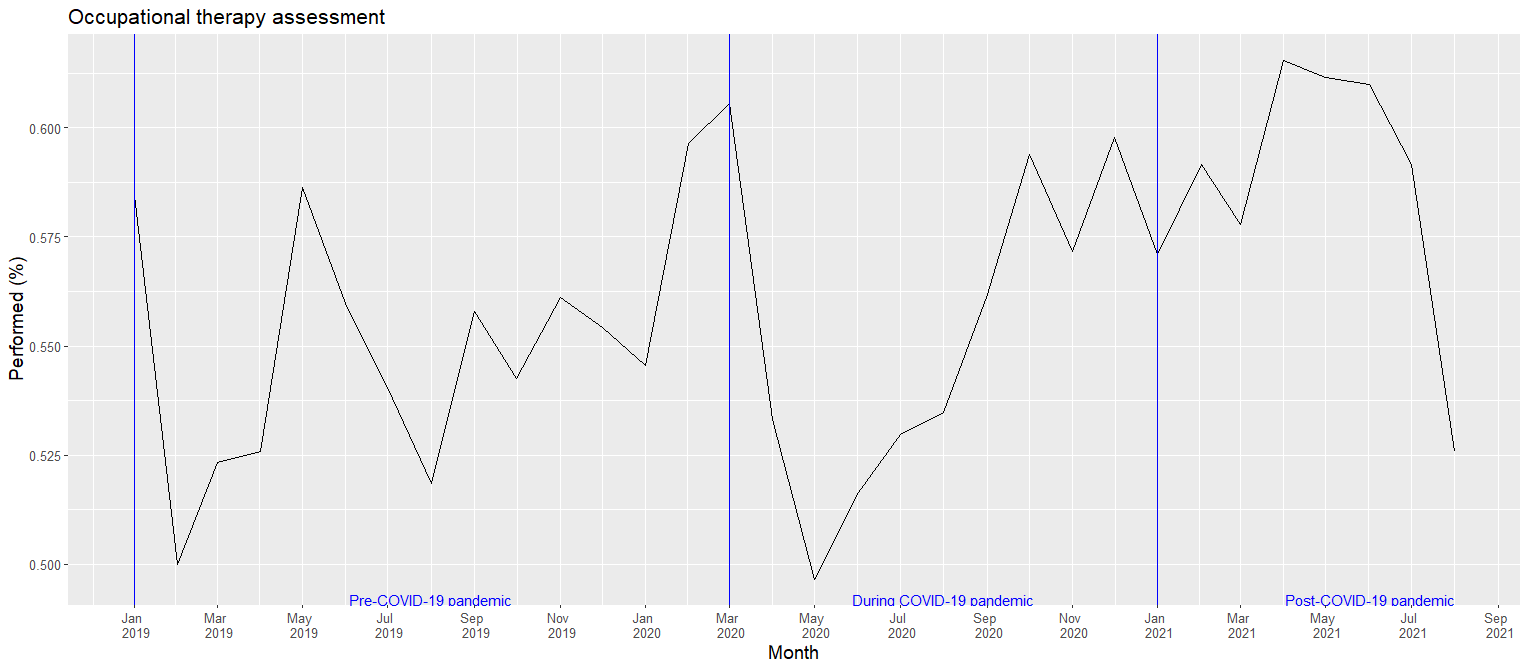


## Supplementary Figure 9. The prescription of cholinesterase inhibitors in the pre-COVID-19, COVID-19 and post-COVID-19 periods


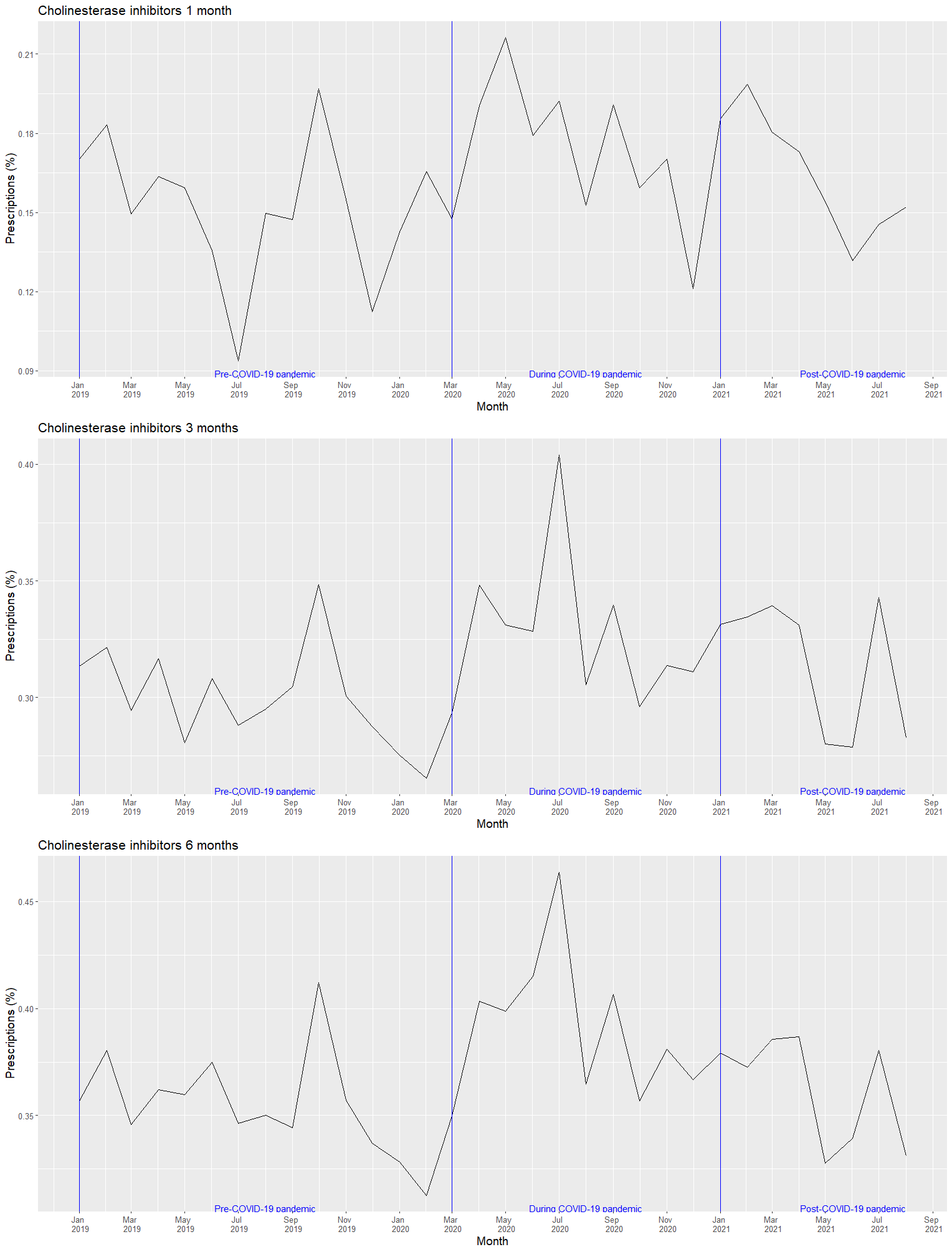


## Supplementary Figure 10. The prescription of memantine in the pre-COVID-19, COVID-19 and post-COVID-19 periods


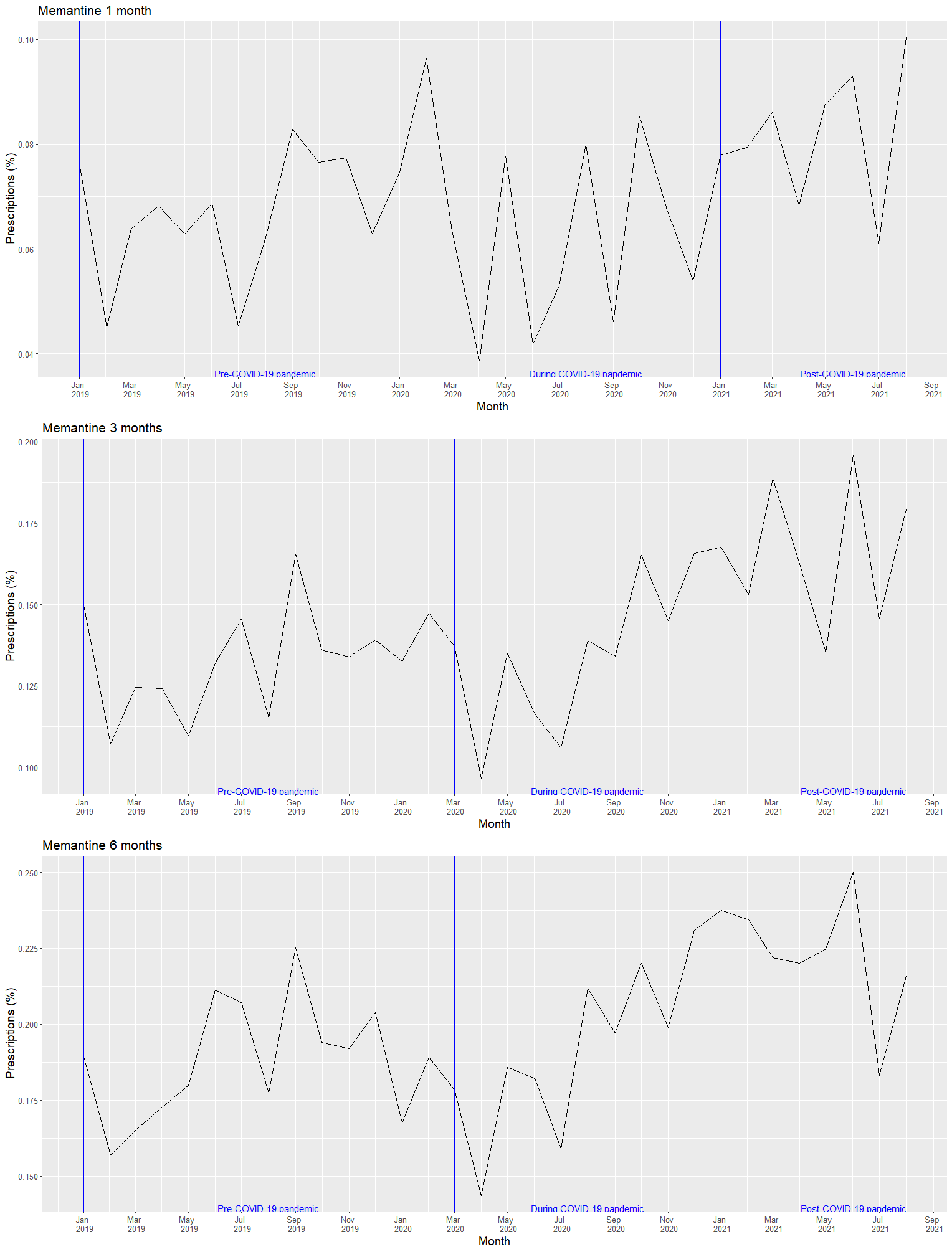


## Supplementary Figure 11. The prescription of antipsychotics in the pre-COVID-19, COVID-19 and post-COVID-19 periods


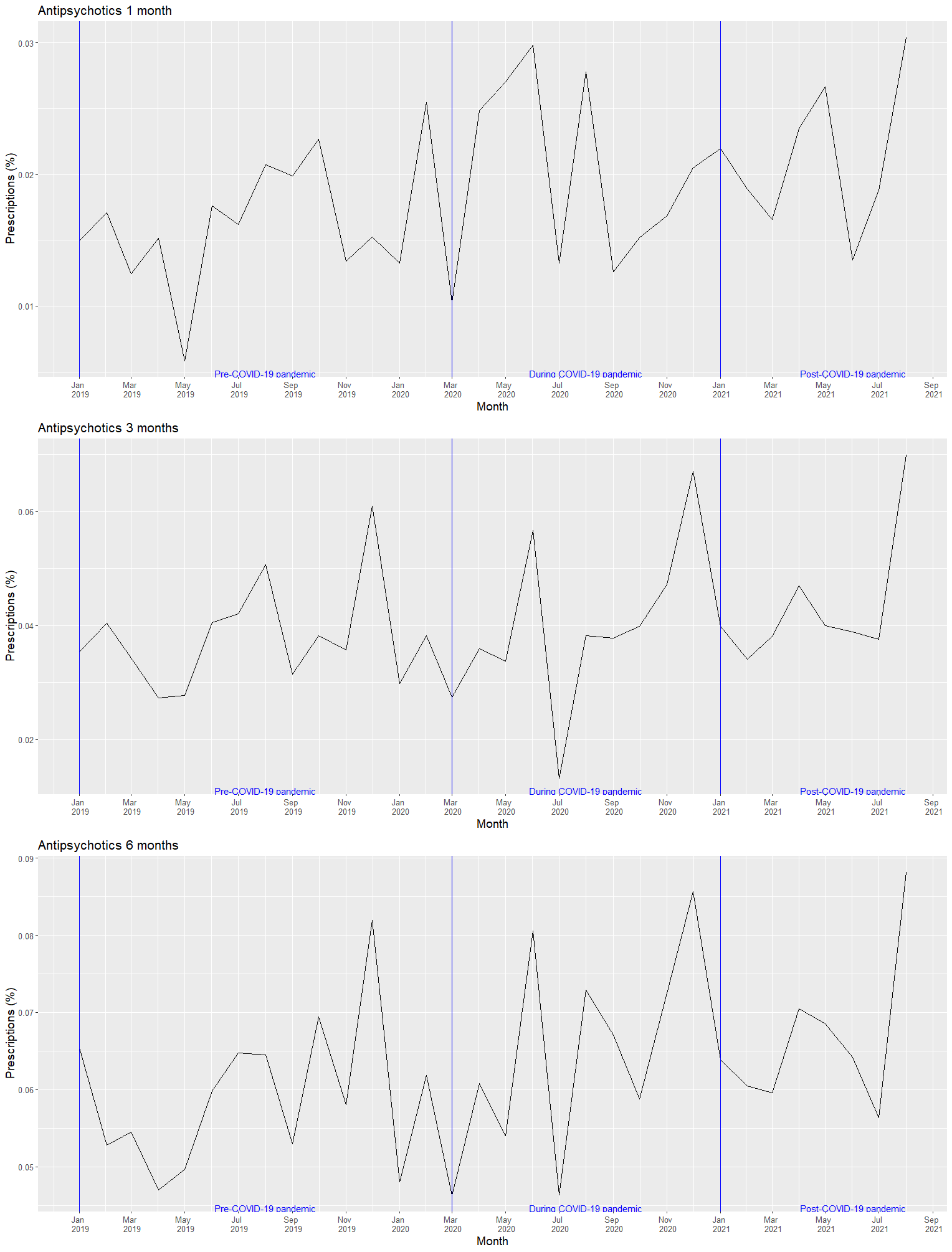


## Supplementary Figure 12. Odds ratio of dementia diagnostics and treatment between the COVID-19 and pre-COVID-19 periods.


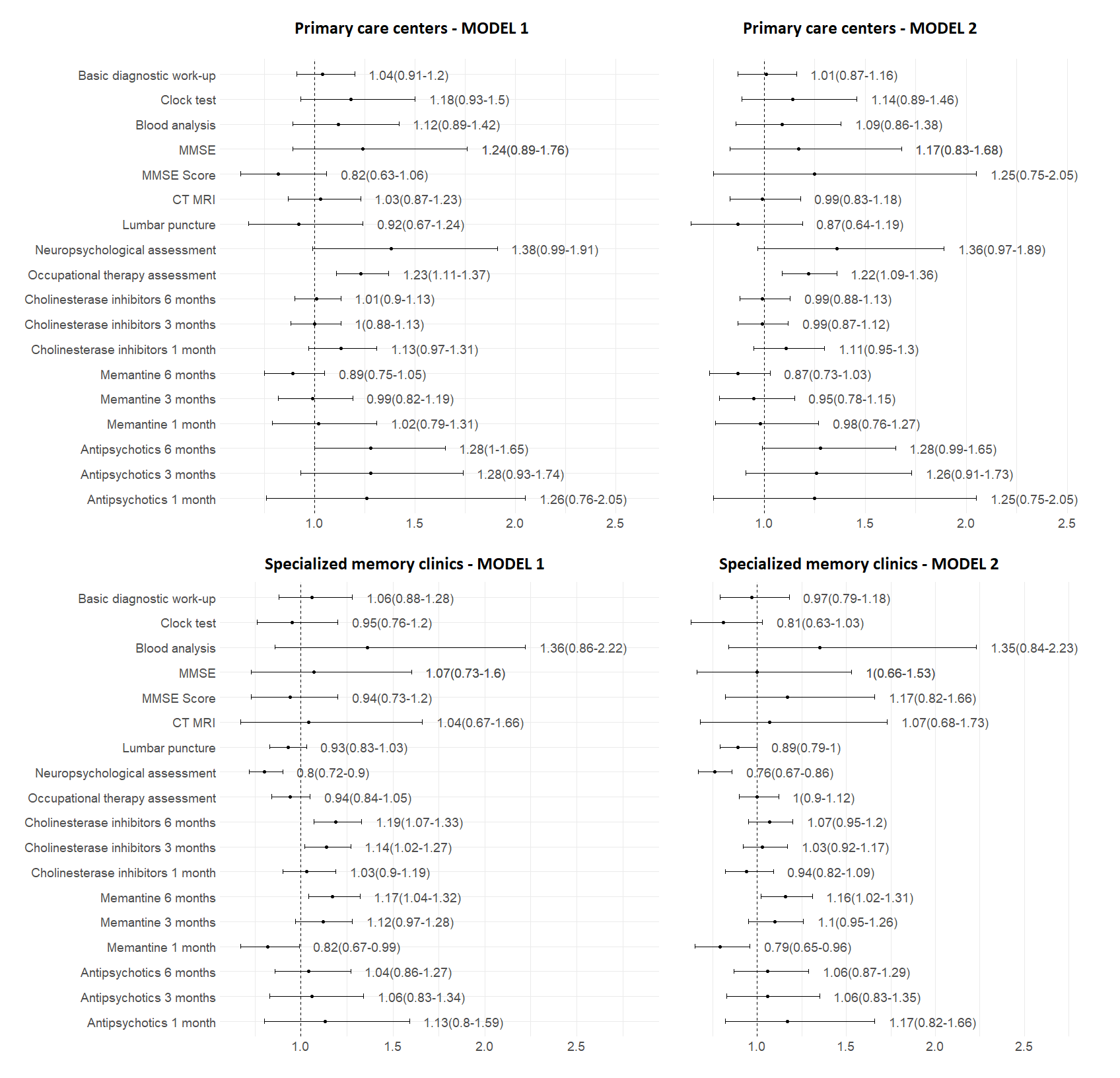


Note: Model 1 was performed with binary logistic regression and controlled for age at the date of dementia diagnosis and sex. Model 2 was additionally adjusted for living areas, cohabitation status, living arrangements, education, individual income, Charlson Comorbidity Index, and types of dementia diagnosis.

## Supplementary Figure 13. Odds ratio of dementia diagnostics and treatment between the post-COVID-19 and pre-COVID-19 periods


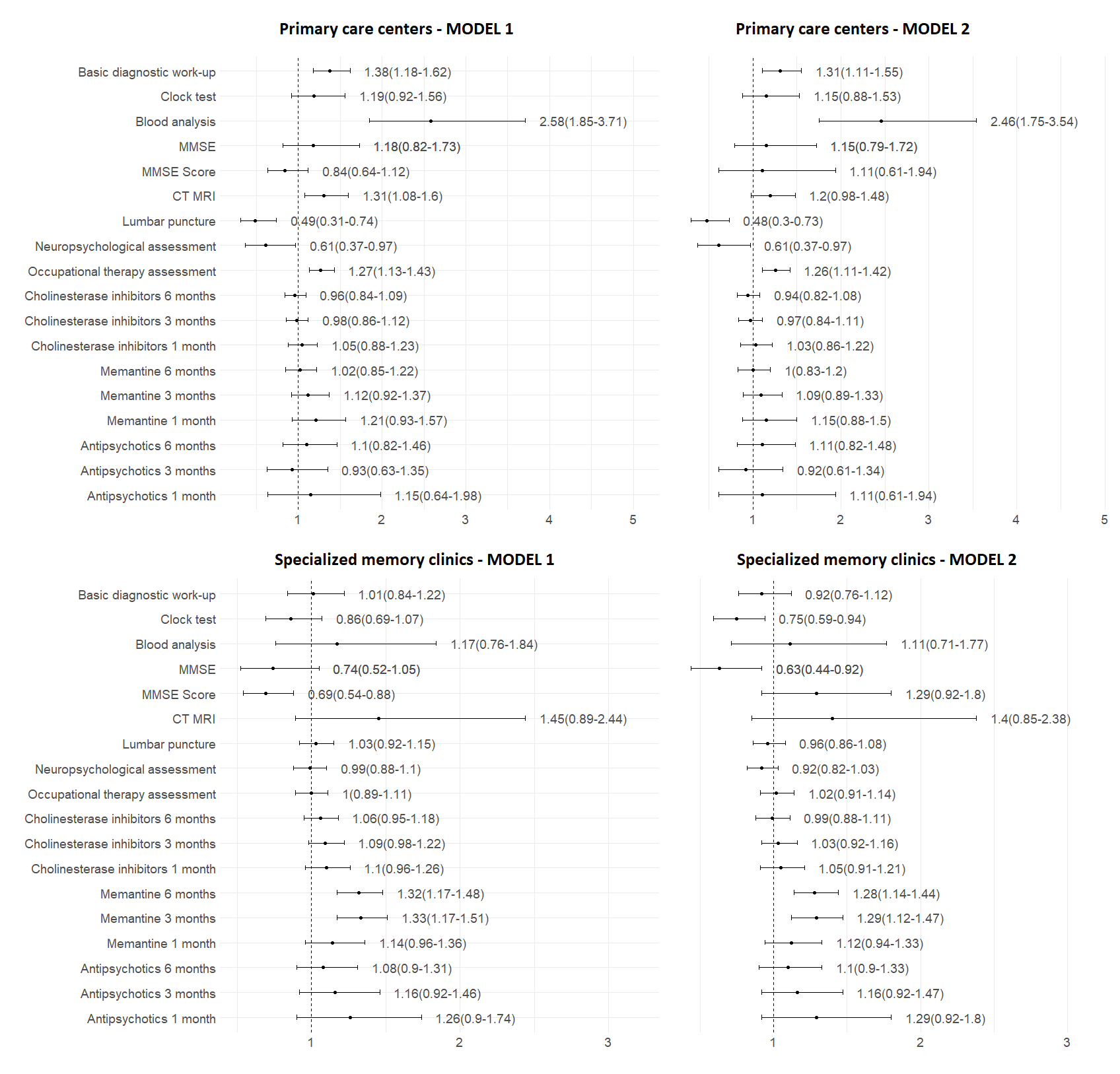


Note: Model 1 was performed with binary logistic regression and controlled for age at the date of dementia diagnosis and sex. Model 2 was additionally adjusted for living areas, cohabitation status, living arrangements, education, individual income, Charlson Comorbidity Index, and types of dementia diagnosis.

## References for Supplementary Material

1. Benchimol EI, Smeeth L, Guttmann A, Harron K, Moher D, Petersen I, et al. The REporting of studies Conducted using Observational Routinely-collected health Data (RECORD) statement. PLoS Med. 2015;12(10):e1001885.

2. Ludvigsson JF, Almqvist C, Bonamy AK, Ljung R, Michaëlsson K, Neovius M, et al. Registers of the Swedish total population and their use in medical research. European journal of epidemiology. 2016;31(2):125-36.

3. Ludvigsson JF, Svedberg P, Olen O, Bruze G, Neovius M. The longitudinal integrated database for health insurance and labour market studies (LISA) and its use in medical research. Eur J Epidemiol. 2019;34(4):423-37.

4. Ludvigsson JF, Andersson E, Ekbom A, Feychting M, Kim JL, Reuterwall C, et al. External review and validation of the Swedish national inpatient register. BMC Public Health. 2011;11:450.

5. Wallerstedt SM, Wettermark B, Hoffmann M. The First Decade with the Swedish Prescribed Drug Register - A Systematic Review of the Output in the Scientific Literature. Basic Clin Pharmacol Toxicol. 2016;119(5):464-9.

6. Wettermark B, Hammar N, Fored CM, Leimanis A, Otterblad Olausson P, Bergman U, et al. The new Swedish Prescribed Drug Register--opportunities for pharmacoepidemiological research and experience from the first six months. Pharmacoepidemiol Drug Saf. 2007;16(7):726-35.

7. Brooke HL, Talbäck M, Hörnblad J, Johansson LA, Ludvigsson JF, Druid H, et al. The Swedish cause of death register. Eur J Epidemiol. 2017;32(9):765-73.

8. The Swedish registry for cognitive/dementia disorders - SveDem. About SveDem Sweden: SveDem; 2022 [updated January 30, 2022; cited 2022 January 30]. Available from: <https://www.ucr.uu.se/svedem/in-english>.
